# Optimization of Alkaline Activator on the Strength Properties of Geopolymer Concrete

**DOI:** 10.3390/polym14122434

**Published:** 2022-06-16

**Authors:** Fatheali A. Shilar, Sharanabasava V. Ganachari, Veerabhadragouda B. Patil, T. M. Yunus Khan, Syed Javed, Rahmath Ulla Baig

**Affiliations:** 1 Department of Civil Engineering, Jain College of Engineering, Belagavi 590014, India; shilarone@gmail.com; 2 Department of Chemistry, School of Advanced Science, KLE Technological University, Hubballi 580031, India; 3 Institute of Energetic Materials, Faculty of Chemical Technology, University of Pardubice, 53210 Pardubice, Czech Republic; iamveerabhadraa@gmail.com; 4 Research Center for Advanced Materials Science (RCAMS), King Khalid University, Abha 61413, Saudi Arabia; yunus.tatagar@gmail.com; 5 Department of Mechanical Engineering, College of Engineering, King Khalid University, Abha 61421, Saudi Arabia; jjaffer@kku.edu.sa; 6 Department of Industrial Engineering, College of Engineering, King Khalid University, Abha 61421, Saudi Arabia; rub786@gmail.com

**Keywords:** geopolymer concrete, red mud, molar ratio, setting time, fresh and mechanical properties

## Abstract

This study investigates the effects of red mud on the performance of geopolymer concrete in regard to fresh and mechanical properties. Red mud was used as a binder, and GGBS replaced the binder. Different proportions of red mud ranging from 0 to 30% with an interval of 2% and activator agents such as KOH and K_2_SiO_3_ for various alkaline-to-binder ratios such as 0.30, 0.40, and 0.50 were used; their effect on the fresh and mechanical properties of geopolymer concrete were the focusing parameter on the current study. Fresh properties such as setting time, slump, compaction factor, and vee-bee consistometer test, and mechanical properties such as compressive strength, split tensile strength, flexural strength, modulus of elasticity, and impact energy were studied. ANOVA and radar plot analysis were studied for various alkaline to binder (A/B) compressive strength results tested for 7 to 90 days. The increase of red mud quantity caused the decline of workability, but there was continuous enhancement of mechanical properties of GPC up to a specific limit. An alkaline-to-binder ratio of 0.4 shows excellent results compared with other ratios at ambient conditions for strength properties. ANOVA and radar plot reveal that A/B of 0.40 for 90 days shows excellent results compared with other ratios, and CS values vary in a linear manner.

## 1. Introduction

In recent years, the sustainable construction of materials has been gaining more attention, especially geopolymer concrete (GPC), which utilizes industrial waste products as binding materials and shows better mechanical properties than OPC [1]. Geopolymer is a primary material composed of an aluminosilicate substance made of a crosslinked structure of AlO_4_ and SiO_4_ [2,3]. It is often manufactured from silicon and aluminum-rich materials, which can stimulate the synthesis of aluminosilicate in a high alkalinity environment [4,5]. When reacting with alkali, fly ash (FA) and ground granulated blast-furnaces slag (GGBS) produce an inorganic polymer binder through polymerization called a geopolymer [6,7]. Factors such as alkaline agents, particle size distribution, and particle size significantly affect the polymerization process [8,9,10]. It was reported that an increase in FA content as binding material increases the CS and substantially impacts the rheology property of GPC. The GPC matrix with industrial waste as binder content decreased carbon emission by 30% to 50% with fly ash and slag [11,12]. Red mud is an appropriate geopolymer precursor due to its high basicity and aluminum content. Much research has been conducted in recent years on a red-mud-based geopolymer. GPC is a clinker-free and low-energy carbon binder [13,14,15]. GPC is prepared through the activation of industrial waste such as fly ash (FA), metakaolin (MK), red mud (RM), and waste glass, as aluminosilicate sources react with alkaline solutions such as alkali activator agents [16]. The latest innovation in sustainability resulted in geopolymer as an alternative construction.

It was using GGBS and red mud with an alkali activator that resulted in the highest compressive (CS) and flexural strengths (FS) [17,18,19]. Numerous papers reported that GGBS-based GPC has concerns with low flowability, rapid setting, high shrinkage, and degradation of mechanical properties after carbonation [20,21,22,23,24]. The blending of FA is more likely than using GGBS to obtain better fresh and hardened properties of GPC. steel fiber and RM are the two additional ingredients for the synthesis of geopolymerization. The regular addition of red mud into a geopolymer solution resulted in a continual loss of intensity and increased widening of the key features in XRD and FTIR spectra for metakaolin-based geopolymer [25,26]. Increasing the amount of red mud in this system resulted in an almost consistent decrease in compressive strength. It used one type of red mud and three different types of fly ash as the source material to create a geopolymer that can be cured at ambient and higher temperatures [27,28,29]. Compared to previous class-F-based geopolymers, it was discovered that compressive strength of 15.2 MPa was attained after an average duration of ambient temperature curing at significantly lower sodium hydroxide content. Sodium silicate and sodium hydroxide activated red-mud-metakaolin-based geopolymer cured for 28 days with a compressive strength of 10 MPa, equivalent water absorption, and density [30,31]. In a geopolymer, many geopolymer products were formed by the interaction of OH with N–A–S–H components in rice husk ash-red mud in a vigorous reaction of nature′s alkali [32].

Various fly ash specimens activated with NaOH 8–12 M and cured at 85 °C for 24 h yielded a material with a compressive strength between 35 and 40 MPa, which increased to approximately 90 MPa when water glass was added to the NaOH (SiO_2_/Na_2_O = 1.23). Furthermore, in such instances, the SiO_2_/Na_2_O ratio is a significant metric, but the water-to-binder ratio must also be considered. Fly ash, kaolinite, sodium silica solution, NaOH, and water are combined to create geopolymers. The compressive strength was impacted by the curing duration and the curing temperature, with samples cured at 60 °C for 48 h producing quality results. The larger molarity of NaOH employed as an alkaline activator proved to produce greater compressive strength while also significantly influencing early strength. A 1:1 mixture of NaOH and sodium silicate (SiO_2_/Na_2_O = 8) was found to activate fly ash geopolymerization and produce outstanding strength development with compressive strength of around 47 MPa. This result is greater than 40 MPa, which is known as the high-strength concrete requirement and confirms the potential of fly ash as a cement alternative.

**Figure 1:**
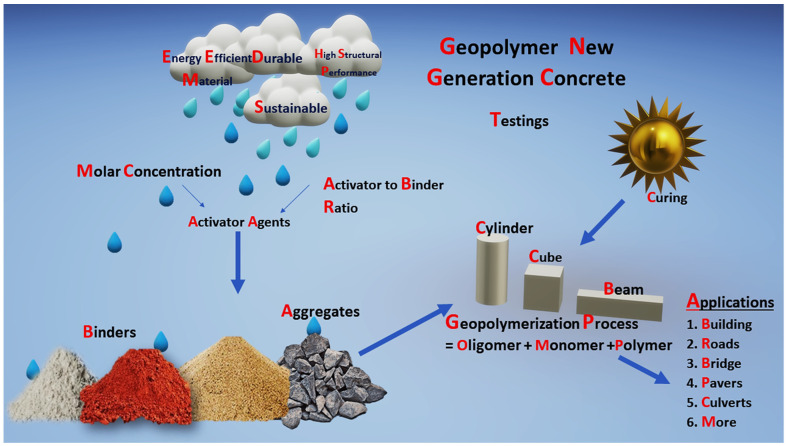
Geopolymerization process and its application.

In GGBS-based GPC, RM was partially replaced with GGBS, and newly formed GPC performances are assessed in the present work. For different A/B ratios of 0.30, 0.40, and 0.50 GPC of KOH and K_2_SiO_3_ activator agent, cubes, cylinders, and beams were cast and tested. GPC was tested for slump, compactor factor, and vee bee consistometer as a part of the workability study. Mechanical properties such as compressive strength, split strength, flexural strength, water absorption, modulus of elasticity, and impact tests were analyzed. Specimens were tested for 7, 14, 28, and 90 days and their results are discussed. ANOVA and radar plot analysis were explored for varied alkaline-to-binder (A/B) ratios of compressive strength results evaluated for 7 to 90 days. Figure 1 shows the stage for preparation of geopolymer concrete and its application.

## 2. Materials Used

Raw materials used to make GPC were GGBS and RM as a binder, and their chemical compositions and physical properties of GPC are shown in Table 1 and Table 2. The size, shape, pore size distribution, and roughness affect particles of specific surface areas. KOH and K_2_SiO_3_ were procured for their study from Suresh Chemicals, Belgaum. For this study, GGBS obtained from Bellary Jindal steel was stored in tight bags, confirmed as per IS 12089, with specific gravity of 2.88. Red mud was procured from Belagavi Karnataka, with specific gravity of 2.68. Locally available river sands and courses were used as aggregates. Fine aggregate has 4.75 mm downsizes with specific gravity as 2.6, fineness modulus as 3, and water absorption as 1%. The coarse aggregate with 20 mm downsizes was used, with specific gravity of 2.8, fineness modulus of 7.0, and water absorption as 1.12%, which was confirmed as per IS: 383, and distilled water was added during the preparation of fresh concrete to improve workability and to make the mix a homogeneous mix.

## 3. Preparation of GPC

**Figure 2:**
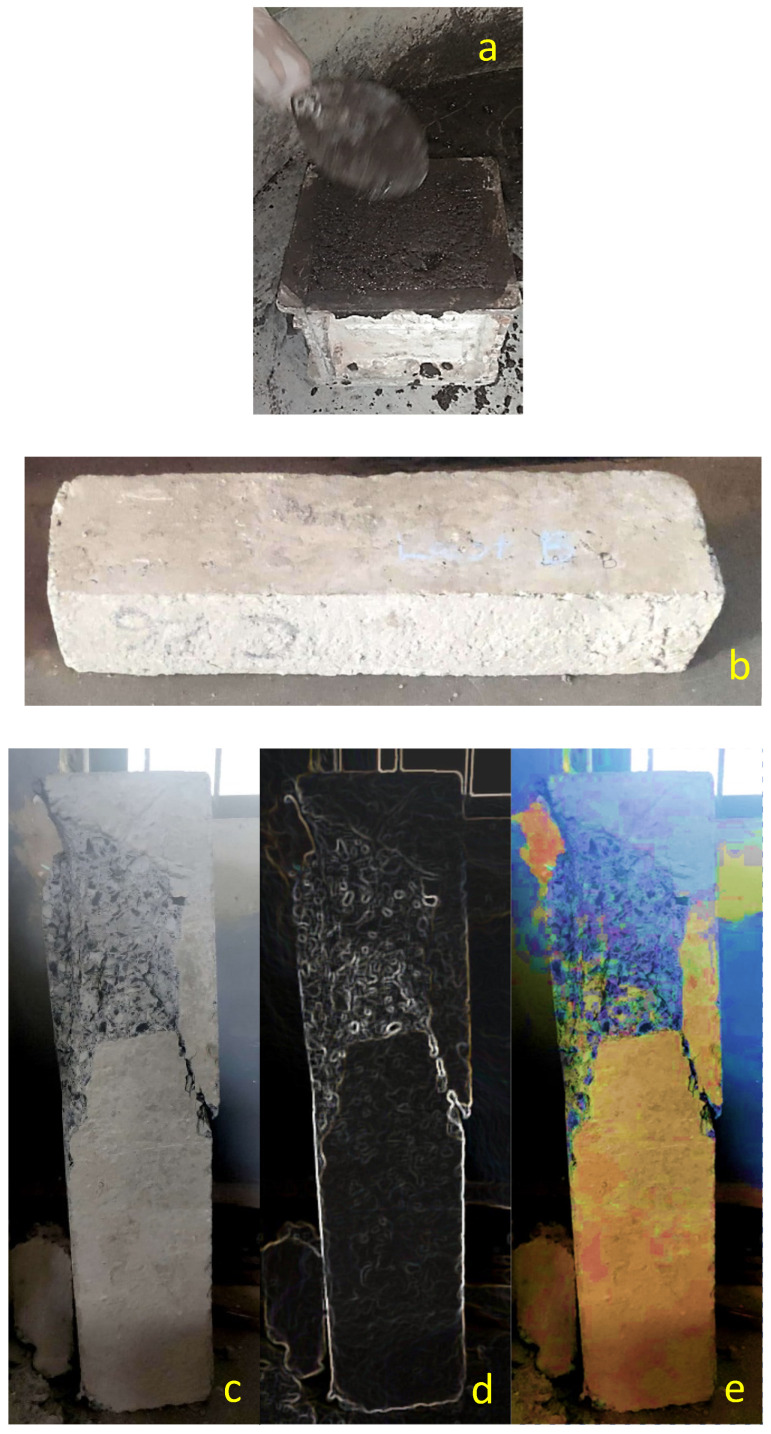
(**a**) Cast of the cube for compressive strength testing. (**b**) Beam specimens before flexural testing. (**c**) Beam specimens after flexural testing. (**d**,**e**) Failure pattern on the surface of the beam.

After classifying the materials required for the preparation of GPC, mix design was prepared after referring to various literature papers and IS code 10262. The basic tests and analysis on all the ingredients of GPC were carried out, i.e., the physical property of procured ingredients of GPC was investigated. KOH was in the pellet form of solid, and they were converted into a liquid by adding considerable water and maintaining the molar concentration of 16 M. The alkaline solution was prepared a day before the casting of GPC. During the day of casting, each ingredient was weighed, dry uniformly mixed, the alkaline solution was mixed with ingredients, and fresh GPC was prepared. The mixing of concrete was carried out through a concrete mixer. The slump (SV), compaction factor (CF), and VB consistometer (VBC) of geopolymer concrete (GPC) were observed during its new state. Casted cubes, cylinders, and beams were tested per IS code. Cube specimens preparation is shown in Figure 2a, beam specimens before testing for 28 days is shown in Figure 2b, failure pattern of beam after 28 days of testing is shown in Figure 2c–e.

Cube of 15 cm^3^, cylinder of 15 × 30 cm^2^, beam of 15 × 15 × 50 mm^3^ were the sizes of specimens cast. Specimens were cured at ambient temperature in the room and covered with bunny bags. Specimens were tested at 7, 14, 28, and 90 days. The mix proportions of GPC are shown in Table 3. CS results tested at 7, 14, 28, and 90 days were used for performing the ANOVA analysis for various A/B ratios of 0.30, 0.40, and 0.50 using the software.

## 4. Setting Time

### 4.1. Initial and Final Setting

**Figure 3:**
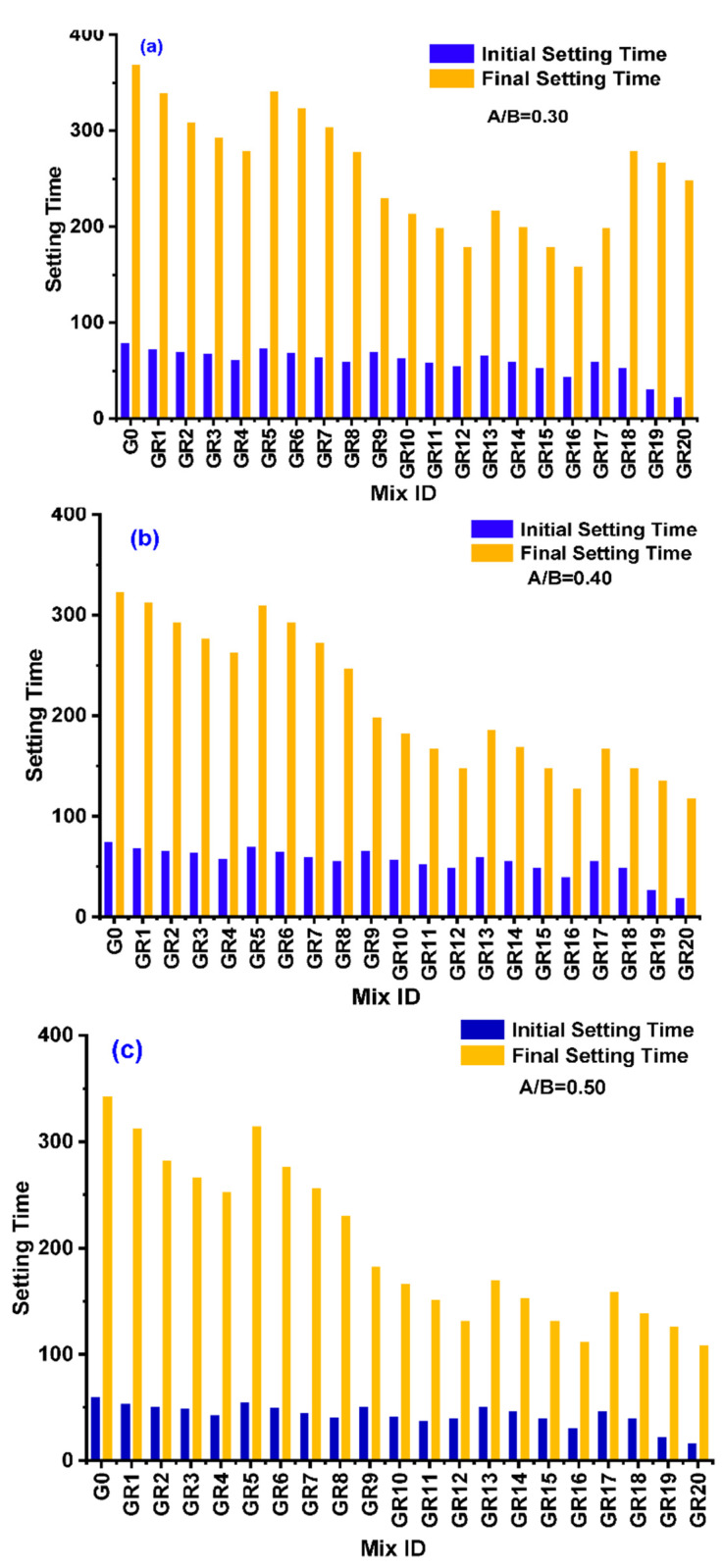
(**a**–**c**) These represent the initial and final setting time for A/B ratio of 0.3, 0.4, and 0.5 for various mixes of GPC.

Geopolymer paste for A/B ratios of 0.30, 0.40, and 0.50 for various mixes from G0 to GS2.0 was prepared, and a setting time test was carried out. Initial and final setting times of geopolymer paste determined by Vicat apparatus were confirmed as per IS code IS 5513. Figure 3a–c represents the initial and final setting time for A/B ratio of 0.3, 0.4, and 0.5 for various mixes of GPC for KOH and K_2_SiO_3_ activator agents.

For the A/B ratio of 0.3 and mix ratio from G0 to GR20, the maximum initial value was observed for the G0 mix (maximum). GR16 was the lowest among the mix, with 155 min and 375 min, respectively. The final setting observed an A/B ratio of 0.3 for the G0 mix (maximum), and GR16 was the lowest among the mix with 80 min and 24 min, respectively. For the A/B ratio of 0.4 and mix ratio from G0 to GR20, the maximum initial values observed for the G0 mix (maximum) and GR16 were the lowest among the mix, with 120 min and 325 min, respectively. With an A/B ratio, 0.3 final settings were observed for the G0 mix (maximum), and GR16 was the lowest among the mix with 75 min and 22 min, respectively. For the A/B ratio of 0.5 and mix ratio from G0 to GR20, the maximum initial value and the final setting were observed for the G0 mix (maximum), and GR16 was the lowest among the mix with 120 min and 350 min, respectively. With an A/B ratio of 0.3, final settings were observed for the G0 mix (maximum), and GR16 was the lowest among the mix, with 64 min and 20 min, respectively. As the A/B ratio increased from 0.3, 0.4, and 0.5, it was observed that the setting time decreased. The A/B ratio of 0.30 showed the maximum setting time compared with other ratios.

### 4.2. Soundness Test

**Figure 4:**
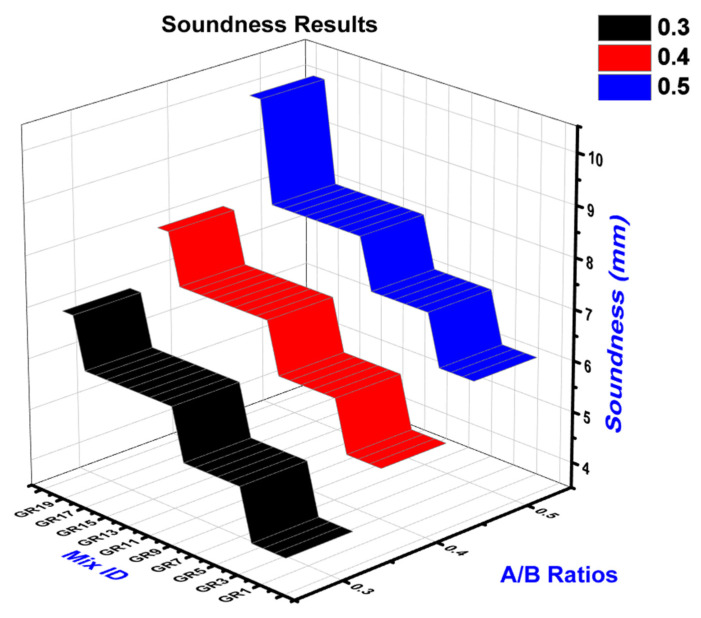
Soundness tests for various A/B ratios.

Soundness tests were carried out as per IS: 4031-PART code. Le Chatelier’s method was used to determine the soundness of geopolymer paste at a room temperature of 29 °C. The soundness results for various A/B ratios of 0.30, 0.40, and 0.50 for KOH and K_2_SiO_3_ as activator agents shown in Figure 4. As the A/B ratio increases, the soundness also increases. A/B ratio of 0.50 shows maximum soundness compared with other ratios. GR19 is the sample for which maximum soundness of 9 mm was observed. GR19 has a maximum RM%, compared with other proportions.

For GR1 with A/B ratios of 0.30, it is recorded as minimum soundness. Expansion might have been caused by the development of reaction chemicals such as ettringite and gypsum. However, because the raw materials include more pozzolana, the available lime and magnesia are utilized in chemical reactions, making the binder sound. The soundness of cementitious materials shall not exceed 10 mm, according to Indian norms. As a result, the FA-GGBS-based geopolymers satisfy the soundness criteria of cementitious materials [12,17,25].

### 4.3. Workability

**Figure 5:**
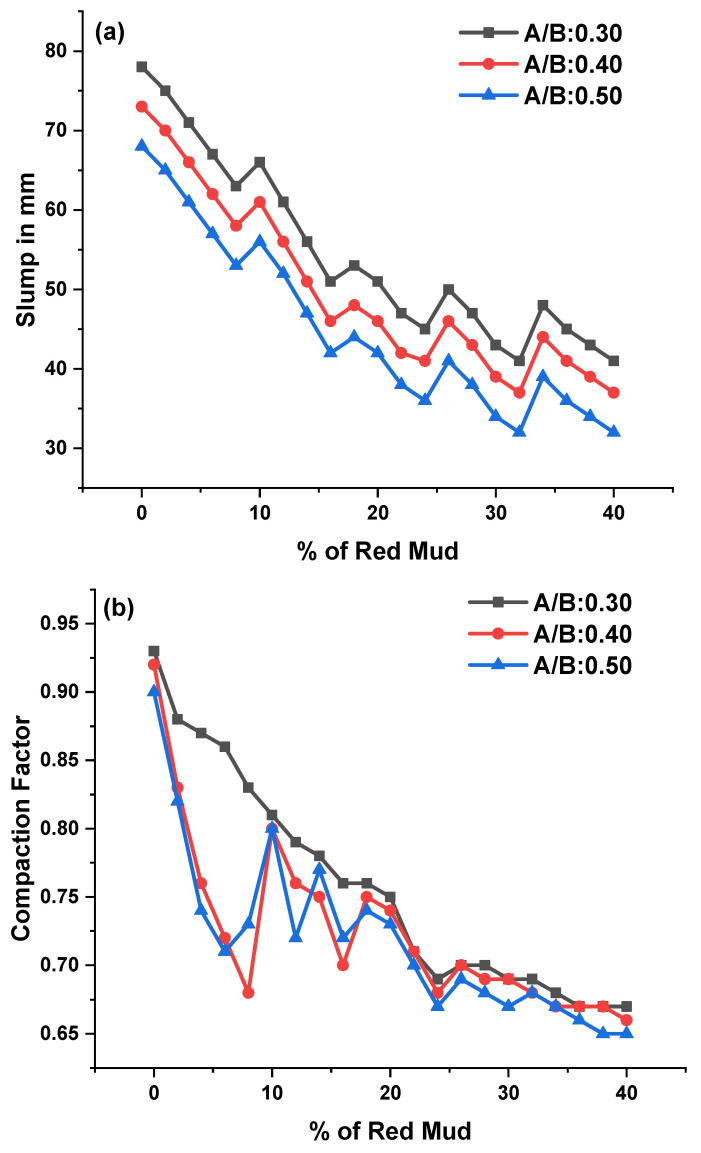

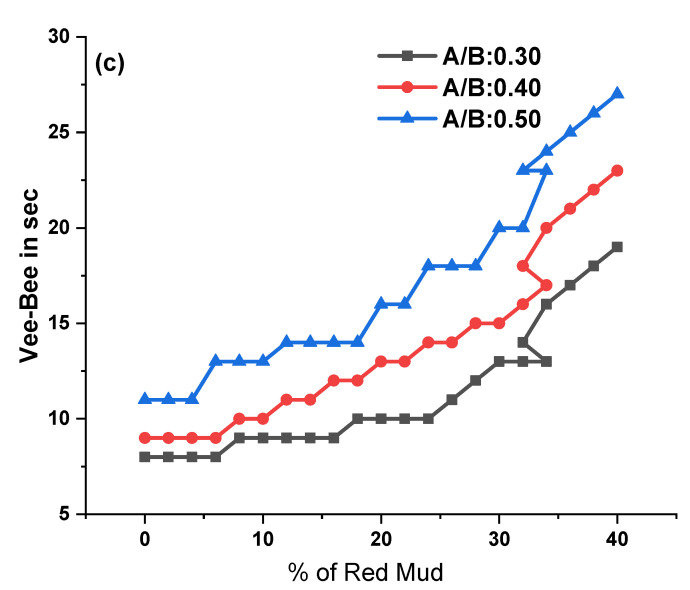
(**a**–**c**) These represent the slump, compaction, and vee-bee time results for A/B ratios of 0.3, 0.4, and 0.5 for various mixes of GPC, respectively.

The workability of GPC with river sand was measured by workability as per IS 1199. Workability factors such as slump (SV), compaction factor (CF), and vee-bee consistometer (VBC) for different proportions of red mud along with various A/B ratios were tested; results are shown in Figure 5.

The SV was observed during experimentation for various GPC-GR series; the range of slump observed was from 34 to 78 mm for various design mixes, represented in Figure 5a for KOH and K_2_SiO_3_ activator agents. For G0, maximum SV was observed at 78 mm, and for GR20 mix, minimum SV was observed at 34 mm for an A/B ratio of 0.30. For the A/B ratio of 0.40, maximum SV was observed as 73 mm for G0, and the minimum was 37 mm for GR20. For the A/B ratio of 0.50, maximum SV was observed as 68 mm for G0, and minimum SV was observed as 34 mm for GR20. As the A/B ratio increased from 0.3 to 0.4 and 0.4 to 0.5, the SV reduced. As the RM content increased, the SV also decreased. The CF was observed during experimentation for various GPC-GR series; the range of CF observed was from 0.66 to 0.92 for various design mixes. 

From Figure 5b, G0, maximum CF was observed at 0.92, and GR20 mix minimum CF was observed at 0.69 for an A/B ratio of 0.30. For the A/B ratio of 0.40 maximum, CF was observed as 0.91 for G0 and minimum CF was observed as 0.68 for GR20. For the A/B ratio, 0.50 maximum CF was observed as 0.90 for G0 and minimum CF was observed as 0.66 for GR20. As the A/B ratio increased from 0.3 to 0.4 and 0.4 to 0.5, the CF decreased. As the RM content increased, the CF also decreased. The VBC was observed during experimentation for various GPC-GR series; the range of VBC observed was from 8 s to 25 s for various design mixes. From Figure 5c, G0, maximum VBC was observed at 15 s, and GR20 mix minimum VBC was observed within 8 s for an A/B ratio of 0.30. For an A/B ratio of 0.30 maximum, VBC was observed as 9 s for G0 and minimum VBC was observed as 21 s for GR20. For an A/B ratio of 0.50 maximum, VBC was observed as 12 s for G0 and minimum VBC was observed as 25 s for GR20. As the A/B ratio increased from 0.3 to 0.4 and 0.4 to 0.5, the CF decreased. As the RM content increased, the CF also decreased.

In comparison to GGBS, RM contains silica and alumina content and has a low heat of hydration, but GGBS has a higher rate of hydration than fly ash, i.e., higher reactivity [33,34]. The shape of particles has a more significant influence on the workability of GPC, insofar as FA particles are spherical. In contrast, GGBS has angular particle shapes, and the larger surface area and high porosity of the rich content of silica-based ingredients are the two factors affecting workability [35,36]. The high amount of amorphous silica with porous structured particles in geopolymer paste increases the specific surface area leading to better reactivity [37,38]. GGBS-based GPC, with increasing fly ash content, increasing the slump and delaying polymerization, was reported after the addition of corncob ash, which is rich in silica content and has a higher specific surface area than GGBS, causing increases in SV [39,40]. The lower water-to-binder ratio was higher in GPC. However, the water content was not sufficient for the reaction, leading to a reduction in the slump. The irregular size and shape of the particle had misled rheology and was distinctive of GPC [12,41]. The ratio of Na_2_SiO_3_ to NaOH increased and then decreased the slump value in lightweight GPC due to the high viscosity of Na_2_SiO_3_, which reduced the flow of mixtures. The aggregate shapes also affected the workability [42,43]. The use of saturated surface dry aggregate led to higher workability. The water inside concrete aggregate was available for a reaction and unreacted particles, leading to increased polymerization [44,45].

### 4.4. Hardened Properties of Geopolymer

#### 4.4.1. Compressive Strength

**Figure 6:**
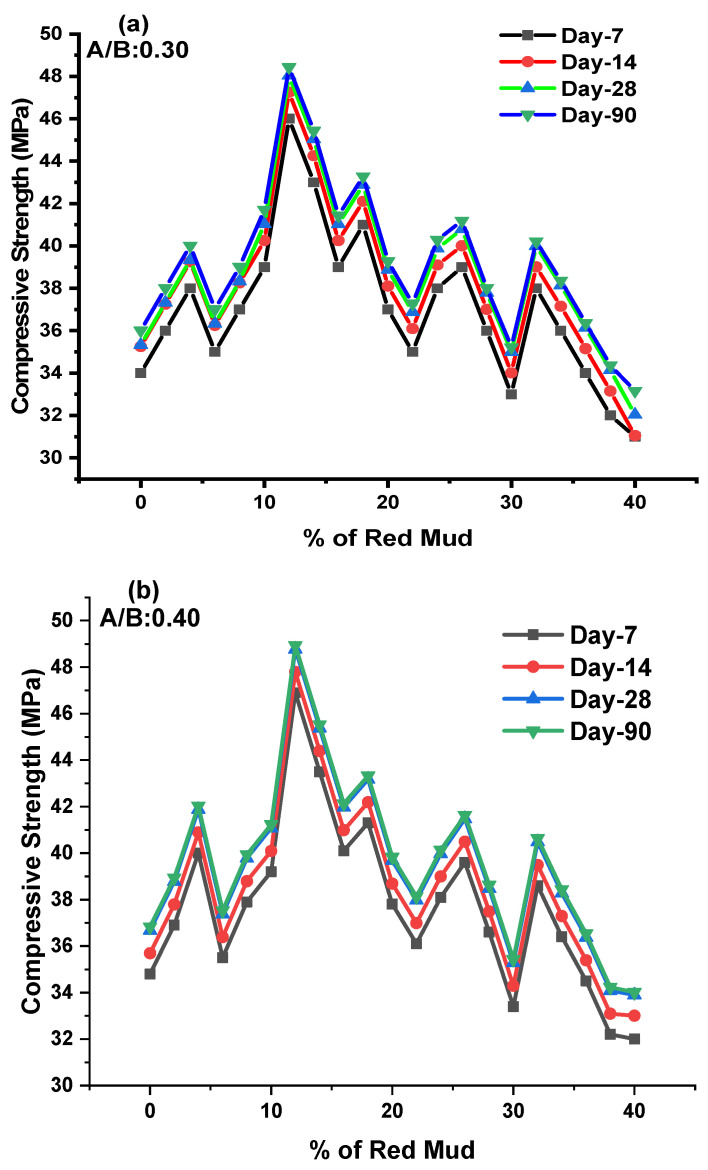

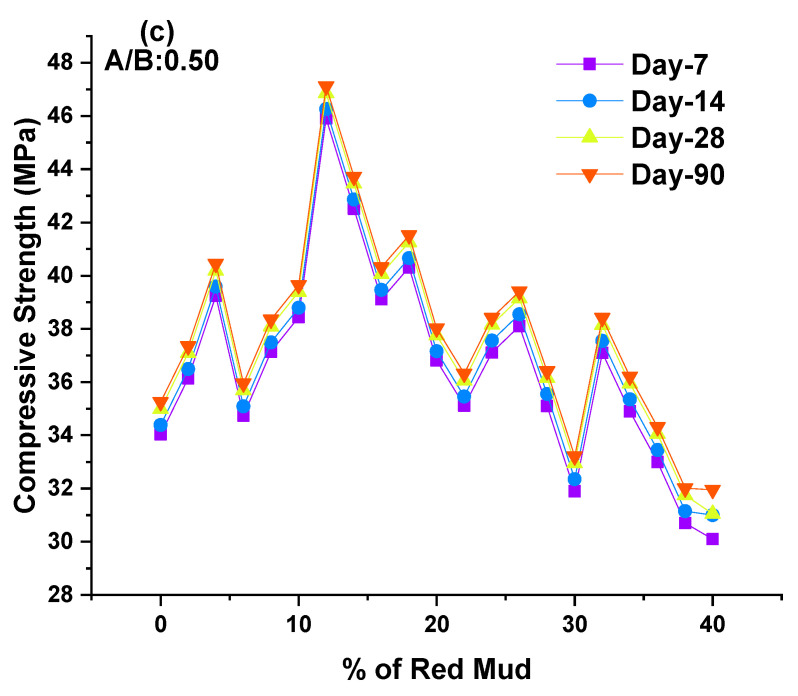
(**a**–**c**) These represent the CS results for 7, 14, 28, and 90 days for A/B ratio of 0.3, 0.4, and 0.5 for various mixes of GPC, respectively.

The compressive strength (CS) test was conducted as per IS 516 code with a 150 mm cube specimen tested at a compression testing machine. The CS valves for various design mixes of geopolymer concrete for the 7, 14, 28, and 90 days are shown in Figure 6a–c.

We obtained results from the CS valve for the GPC-GR series on the 7, 14, 28, and 90 days of testing at ambient condition. The CS was observed during experimentation for various GPC-GR series; the range of CS observed was from 31 to 48.45 MPa for various design mixes, with an A/B ratio of 0.30 represented in Figure 6a for KOH and K_2_SiO_3_ activator agents.

For day 7, maximum CS was observed at 47 MPa for the GR6 mix, and minimum CS was observed at 31 MPa for the GR20 mix. For day 14, maximum CS was observed at 47.25 MPa for the GR6 mix, and minimum CS was observed at 31.5 MPa for the GR20 mix. For day 28, maximum CS was observed at 48.10 MPa for the GR6 mix, and minimum CS was observed at 32 MPa for the GR20 mix. For day 90, maximum CS was observed at 48.45 MPa for the GR6 mix, and minimum CS was observed at 33.16 MPa for the GR20 mix. The CS was observed during experimentation for various GPC-GR series; the range of CS observed was from 32 to 48.93 MPa for various design mixes, with an A/B ratio of 0.40 represented in Figure 6b. For day 7, maximum CS was observed at 46.9 MPa for the GR6 mix, and minimum CS was observed at 32 MPa for the GR20 mix. For day 14, maximum CS was observed at 47.79 MPa for the GR6 mixes, and minimum CS was observed at 33 MPa for the GR20 mixes. For day 28, maximum CS was observed at 48.79 MPa for the GR6 mix, and minimum CS was observed at 33.9 MPa for the GR20 mixes. For day 90, maximum CS was observed at 48.93 MPa for the GR6 mixes, and minimum CS was observed at 34.01 MPa for the GR20 mixes.

The CS was observed during experimentation for various GPC-GR series; the range of CS observed was from 30.1 to 47.12 MPa for various design mixes, with an A/B ratio of 0.50 represented in Figure 6c. For day 7, maximum CS was observed at 45.91 MPa for the GR6 mix, and minimum CS was observed at 30.1 MPa for the GR20 mix. For day 14, maximum CS was observed at 46.26 MPa for the GR6 mix, and minimum CS was observed at 31 MPa for the GR20 mix. For day 28, maximum CS was observed at 46.86 MPa for the GR6 mix, and minimum CS was observed at 31.05 MPa for the GR20 mix. For day 90, maximum CS was observed at 47.12 MPa for the GR6 mix, and minimum CS was observed at 31.95 MPa for the GR20 mix. As the percentage of RM increased up to 12%, the CS was found to be at maximum; beyond 12%, the CS declined. The optimum dosage of RM of 12% maximum CS was observed for 7, 14, 28, and 90 days for an A/B ratio of 0.40. For various ratios of A/B, the 0.40 shows higher results than other ratios.

The GGBS is the majority content among other binding materials. The GGBS has higher specific surface areas, which tend to have a higher heat of hydration, which helps to gain the strength of concrete [24,46,47,48]. It is observed that CS reduces beyond RM 12% after addition in GPC. Na_2_SiO_3_ solution as an alkaline solution result in more silica gel from GGBS and RM subsidizes denser Si–O–Si bonds during polymerization [49,50,51]. However, the Si–O–Al bond is considerably more resilient than Si–O–Si and Al bonds, leading to higher compressive strength [52,53,54]. NaOH solution detaches the silica and alumina present in the mixture as binding agents, endorses the monomer bond structure, and enhances the geopolymerization process. Si/Al ratio is 2, higher CS is achieved, and Si/Al ratio is beyond 4.17 [18,55,56]. The reduction in compressive strength of silica-rich materials adversely affects the matrix structure of the geopolymer composite, which causes the formation of silica gel to be hindered—excess of silicate delays evaporation of water during the polycondensation process [57,58,59]. GGBS content is increased in GPC. The CS also increases due to the aluminosilicate glassy nature of GGBS. When it reacts with alkaline activators and is dissolved in it, and calcium content increases in GPC, it increases the strength and reduces the rate of workability [60,61,62]. RM substitution with GWS in minimal amounts improves cement particle dispersion in the mix, resulting in improved cement reactions and, ultimately, increases in strength and other concrete properties [10,59,63]. The GWS increase in reactive phases implies that the alkali fusion process resulted in physicochemical changes such as the breakage of specific crystal structures and the liberation of silica and alumina, which enhanced reactivity, leading to increased CS [48,64,65]. Compressive strength dropped as the K/Al ratio increased. The exception was a local maximum at a K/Al ratio of one, significant at laboratory temperature and after 200 °C exposure. The local maximum dropped with increasing exposure temperatures, and it was no longer visible between 1000 °C and 1200 °C. The pattern of increasing compressive strength was followed by a decline with increasing Na/Al ratios [66,67,68]. The presence of silica and free lime in the RM, which enhances C–A–S–H gel formation, is the foundation for increased strength achievement. With a high degree of RM replacement, an incomplete geopolymeric reaction was discovered; due to insufficient alkaline content, the geopolymer concrete strength was reduced because the presence of dissolved “Si” and “Al” created more sodium aluminosilicate gel [66,67,68].

#### 4.4.2. Split Tensile Strength and Flexural Strength

**Figure 7:**
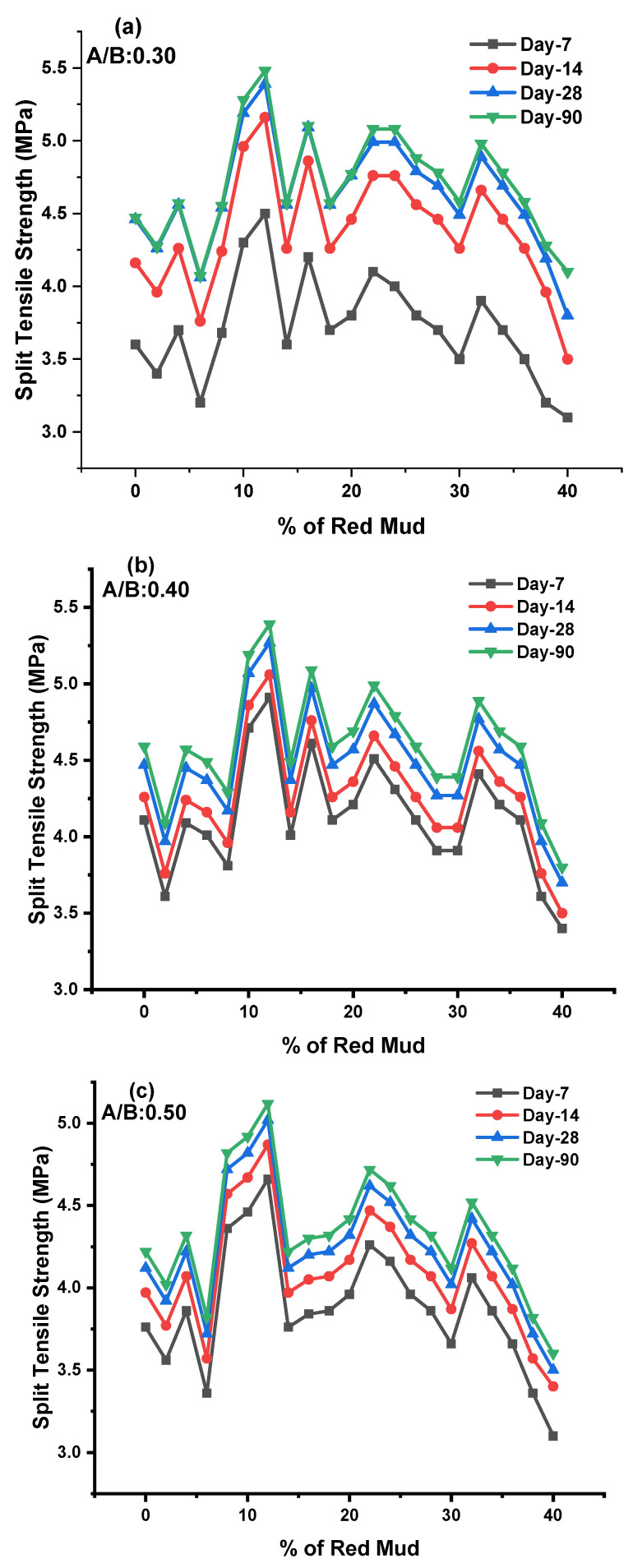
(**a**–**c**) These represent the STS results for 7, 14, 28, and 90 days for A/B ratio of 0.3, 0.4, and 0.5 for various mixes of GPC, respectively.

The split tensile strength (STS) test was conducted as per IS 5816 code in a compression testing machine with a 150 × 300 mm^2^ cylinder specimen. The STS valves for various design mixes of geopolymer concrete for the 7, 14, 28, and 90 days are shown in Figure 7a–c for KOH and K_2_SiO_3_ activator agents.

We obtained results from the STS valve for the GPC-GR series on the 7, 14, 28, and 90 days of testing at ambient condition. The STS observed during experimentation for various GPC-GR series ranged from 3.1 to 5.48 MPa for various design mixes, with an A/B ratio of 0.30 represented in Figure 7a. For day 7, maximum STS was observed at 4.5 MPa for the GR10 mix, and minimum STS was observed at 3.1 MPa for the GR20 mix. For day 14, maximum STS was observed at 5.16 MPa for the GR10 mix, and minimum STS was observed at 3.5 MPa for the GR20 mix. For day 28, maximum STS was observed at 5.39 MPa for the GR10 mix, and minimum STS was observed at 3.8 MPa for the GR20 mix. For day 90, maximum STS was observed at 5.49 MPa for the GR10 mix, and minimum STS was observed at 4.1 MPa for the GR20 mix.

The STS observed during experimentation for various GPC-GR series ranged from 3.4 to 5.39 MPa for various design mixes, with an A/B ratio of 0.40 represented in Figure 7b. For day 7, maximum STS was observed at 4.91 MPa for the GR10 mix, and minimum STS was observed at 3.4 MPa for the GR20 mix. For day 14, maximum STS was observed at 5.06 MPa for the GR10 mix, and minimum STS was observed at 3.5 MPa for the GR20 mix. For day 28, maximum STS was observed at 5.27 MPa for the GR10 mix, and minimum STS was observed at 3.7 MPa for the GR20 mix. For day 90, maximum STS was observed at 5.39 MPa for the GR6 mix, and minimum STS was observed at 3.8 MPa for the GR20 mix.

The STS observed during experimentation for various GPC-GR series ranged from 3.1 to 5.12 MPa for various design mixes, with an A/B ratio of 0.50 represented in Figure 7c. For day 7, maximum STS was observed at 4.66 MPa for the GR10 mix, and minimum STS was observed at 3.1 MPa for the GR20 mix. For day 14, maximum STS was observed at 4.87 MPa for the GR10 mix, and minimum STS was observed at 3.4 MPa for the GR20 mixes. For day 28, maximum STS was observed at 5.02 MPa for the GR10 mix, and minimum STS was observed at 3.5 MPa for the GR20 mixes. For day 90, maximum STS was observed at 5.12 MPa for the GR10 mix, and minimum STS was observed at 3.6 MPa for the GR20 mixes. As the percentage of RM increased up to 20%, the STS was found to be at maximum; beyond 20%, the STS declined. The optimum dosage of RM of 20% maximum STS was observed for 7, 14, 28, and 90 days, for an A/B ratio of 0.40. For various ratios of A/B, the 0.40 shows remarkable results compared with other ratios.

**Figure 8:**
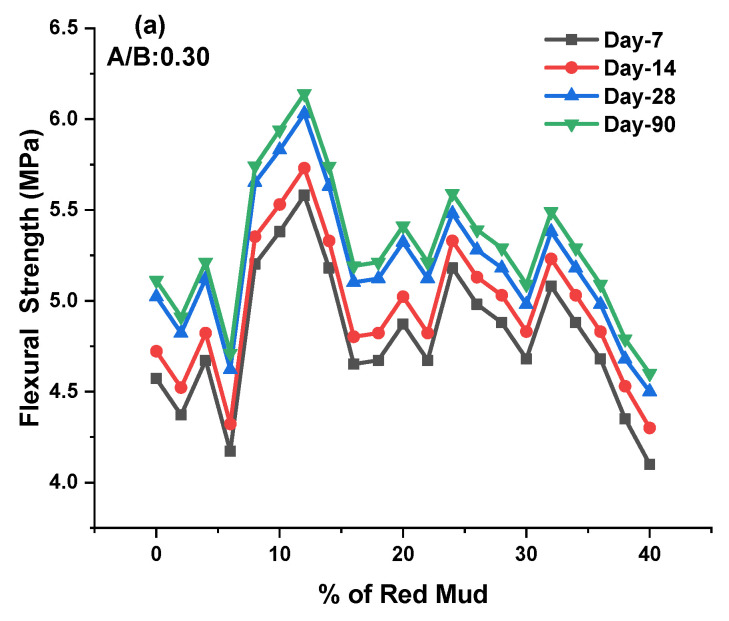

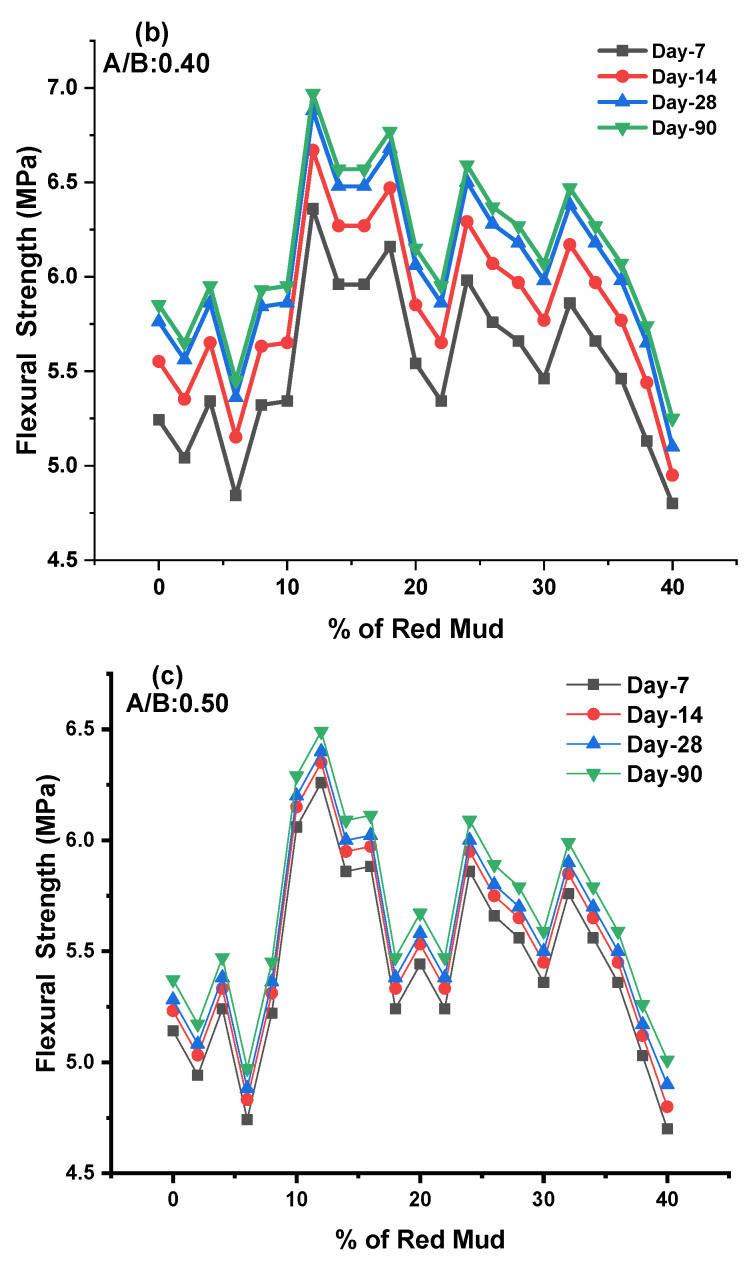
(**a**–**c**) These represent the FS results for 7, 14, 28, and 90 days for A/B ratio of 0.3, 0.4, and 0.5 for various mixes of GPC, respectively.

The flexural strength (FS) test was conducted as per IS 516 code in a compression testing machine with a beam mold (10 × 10 × 50) cm³ specimen. The FS valves for various design mixes of geopolymer concrete for the 7, 14, 28, and 90 days are shown in Figure 8a–c. We obtained results from the FS valve for the GPC-GR series on the 7, 14, 28, and 90 days of testing at ambient conditions. The FS was observed during experimentation for various GPC-GR series; the range of FS observed was from 4.1 to 6.14 MPa for various design mixes, with an A/B ratio of 0.30 represented in Figure 8a.

For day 7, maximum FS was observed at 5.58 MPa for the GR10 mix, and minimum FS was observed at 4.1 MPa for the GR20 mix. For day 14, maximum FS was observed at 5.73 MPa for the GR10 mix, and minimum FS was observed at 4.3 MPa for the GR20 mix. For day 28, maximum FS was observed at 6.03 MPa for the GR10 mix, and minimum FS was observed at 4.5 MPa for the GR20 mix. For day 90, maximum FS was observed at 6.14 MPa for the GR10 mix, and minimum FS was observed at 4.6 MPa for the GR20 mix.

The FS was observed during experimentation for various GPC-GR series; the range of FS observed was from 4.8 to 6.97 MPa for various design mixes, with an A/B ratio of 0.40 represented in Figure 8b. For day 7, maximum FS was observed at 6.36 MPa for the GR10 mix, and minimum FS was observed at 4.8 MPa for the GR20 mix. For day 14, maximum FS was observed at 6.67 MPa for the GR10 mixes, and minimum FS was observed at 4.95 MPa for the GR20 mix. For day 28, maximum FS was observed at 6.88 MPa for the GR10 mix, and minimum FS was observed at 5.1 MPa for the GR20 mix. For day 90, maximum FS was observed at 6.97 MPa for the GR6 mix, and minimum STS was observed at 5.25 MPa for the GR20 mix.

The FS was observed during experimentation for various GPC-GR series; the range of FS observed was from 4.7 to 6.49 MPa for various design mixes, with an A/B ratio of 0.50 represented in Figure 8c. For day 7, maximum FS was observed at 6.26 MPa for the GR10 mix, and minimum FS was observed at 4.7 MPa for the GR20 mix. For day 14, maximum FS was observed at 6.35 MPa for the GR10 mix, and minimum FS was observed at 4.8 MPa for the GR20 mix. For day 28, maximum FS was observed at 6.4 MPa for the GR10 mix, and minimum FS was observed at 4.9 MPa for the GR20 mix. For day 90, maximum FS was observed at 6.49 MPa for the GR10 mix, and minimum FS was observed at 5.01 MPa for the GR20 mix. As the percentage of RM increased up to 20%, the FS was found to be at maximum; beyond 20%, the STS declined. The optimum dosage of RM of 20% maximum FS was observed for 7, 14, 28, and 90 days for an A/B ratio of 0.40. For various ratios of A/B, the 0.40 shows higher results than other ratios.

The size, shape, and type of aggregate, the bond between binding agent and aggregate, and the bonding strength of geopolymer gel play a significant role in developing the split strength [7,69,70,71]. It was reported that the binding strength of geopolymer gel is interrelated with the high level of dissolution of aluminosilicates in alkaline agent presences, leading to increased geopolymerization. The solubility rate is different for GGBS and other silica-content-rich ingredients. The RM, up to 18% in the overall mix, leads to an increase in STS. Beyond 18%, there is external impurity in the granite dust, reducing strength [47,52,72]. With increasing K/Al ratios, compressive and flexural strength and modulus of elasticity dropped. The exception was the local maximum at K/Al ratio 1, which was most visible at laboratory and 200 °C temperatures. Compressive strength testing in situ was not comparable to compressive strength testing after exposure to increased temperatures, which followed a diminishing course with increasing temperature. From 600 °C, the compressive strength values measured in situ began to grow. The average pore width grew marginally with increasing potassium concentration in the laboratory and at raised temperatures up to 800 °C, but fell dramatically from 1000 °C to 1200 °C. The effect of the K/Al ratio on total pore volume was not statistically significant [53,56,73,74].

The interlocking between binding agent and aggregate was insufficient and resulted in earlier decreases in strength. GGBS was partially replaced with rice husk ash. After experimentation, the maximum tensile strength observed was 7.33 MPa at 90 days for 15% rice husk ash. It was reported that the appearance of fractures in a matrix is most likely due to the escape of free water that did not participate in the reaction [47,73,74,75]. With increased Na content, fractures in GPC were reduced. This might be due to a more soluble silicate phase (with increased Na concentration) that functioned as a filler and was dried into fractures, reducing porosity. The existence of multiple crystalline mineral phases, particle size distribution, and form of RM particles—these all parameters may contribute to the microstructures variability [42,76,77]. Geopolymerization has been demonstrated to occur at the surface of aluminosilicate particles. As the molar concentration increases, the FS increases for ambient curing conditions. A more viscous activator agent results in a decrease in the unreacted particle of GGBS in the matrix [19,54,78]. Due to this, there is strong bond development between silica and alumina ions. K_2_SiO_3_/KOH ratio is decreased, making sodium silicate less viscous than sodium hydroxide when decreased, resulting in the decrement of FS [79,80].

#### 4.4.3. Water Absorption and Bulk Density

**Figure 9:**
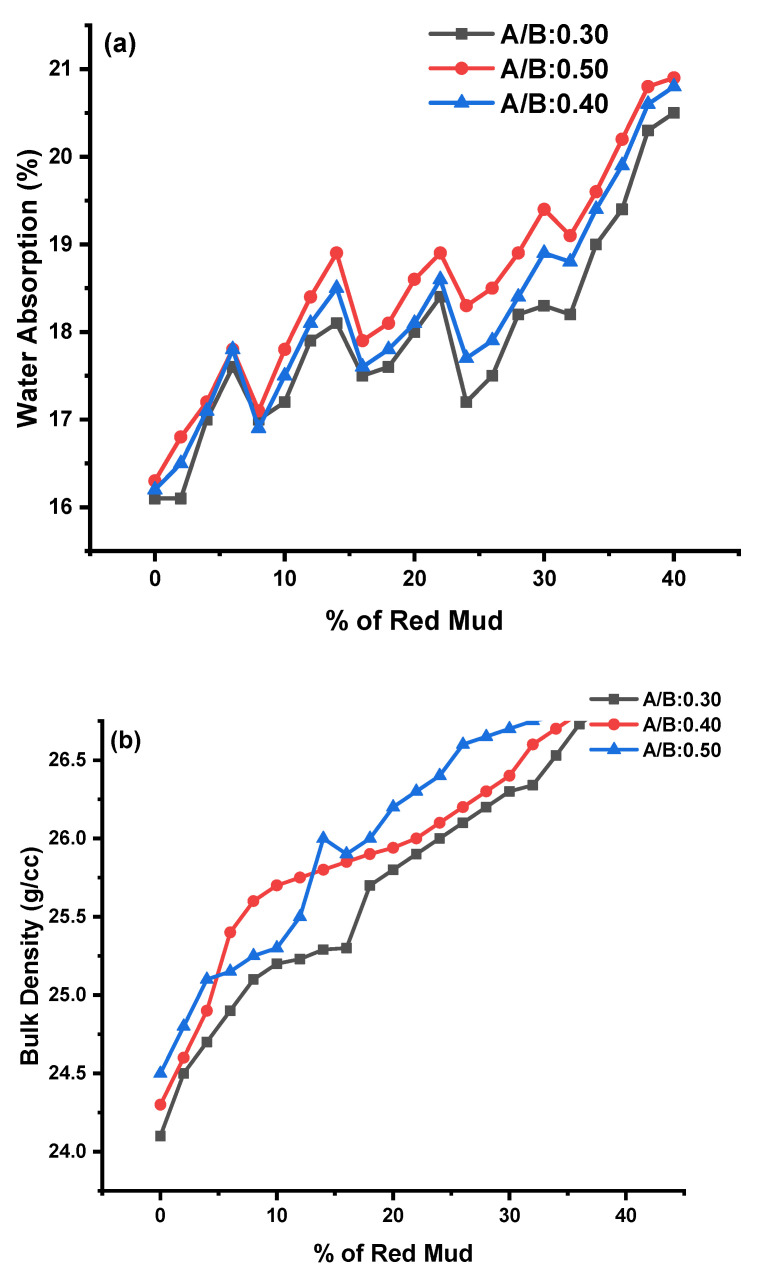
(**a,b**) These represent the WA and BD results for A/B ratios of 0.3, 0.4, and 0.5 for various mixes of GPC, respectively.

The water absorption (WA) test for GPC was carried out as per the c1585 code. GPC prepared with an A/B ratio of 0.40 had slightly higher WA than 0.30 and 0.40. GR20 had maximum WA with 20.5%, 20.9%, and 20.8% for A/B ratio of 0.30, 0.40, and 0.50, respectively. GR8 had minimum WA with 17.5%, 17.9%, and 17.6% for A/B ratio of 0.30, 0.40, and 0.50, respectively, as shown in Figure 9a. Bulk density (BD) of GPC was in the range of 24.1 to 26.4 g/cc. GR20 had maximum WA with 25.6%, 26.4%, and 26.4% for A/B ratio of 0.30, 0.40, and 0.50, respectively. GR8 had minimum WA with 24.1%, 24.3%, and 24.5% for A/B ratios of 0.30, 0.40, and 0.50, respectively, as shown in Figure 9b for KOH and K_2_SiO_3_ activator agents.

The enhancement in GPC properties is a cause of the increasing alkaline-to-binder (A/B) ratio. The increase in the A/B ratio increases the content of Si because the activator contained sodium silicate, which led to an enhancement in the SiO_2_/Al_2_O_3_ in the matrix and made Si-O-Si bonds stronger. This results in a denser matrix of GPC being achieved [81,82,83]. Water ejected from the geopolymer matrix during heat curing creates discontinuous nanopores inside the matrix, increasing geopolymer strength. However, not all moisture will be evacuated from the geopolymer matrix, especially in bigger specimens. Large specimens with higher surface tensions tend to release retained moisture slower than smaller ones. The most likely source of temperature resistance variation related to size is a mixture of these two tendencies. After 400 °C exposure, the Na-based fly ash geopolymer showed more weight loss in TGA and a more prominent DTA peak at around 100 °C than its K-based counterpart. The higher weight loss and DTA peak are associated with the loss of absorbed and combined water in geopolymer gels, indicating that more geopolymer gels are retained in the Na-based geopolymer after 400 °C exposure than in the K-based system, which is consistent with the observed compressive strength results. After being exposed to 800 °C, the tendency reverses.

#### 4.4.4. Modulus of Elasticity (MoE)

**Figure 10:**
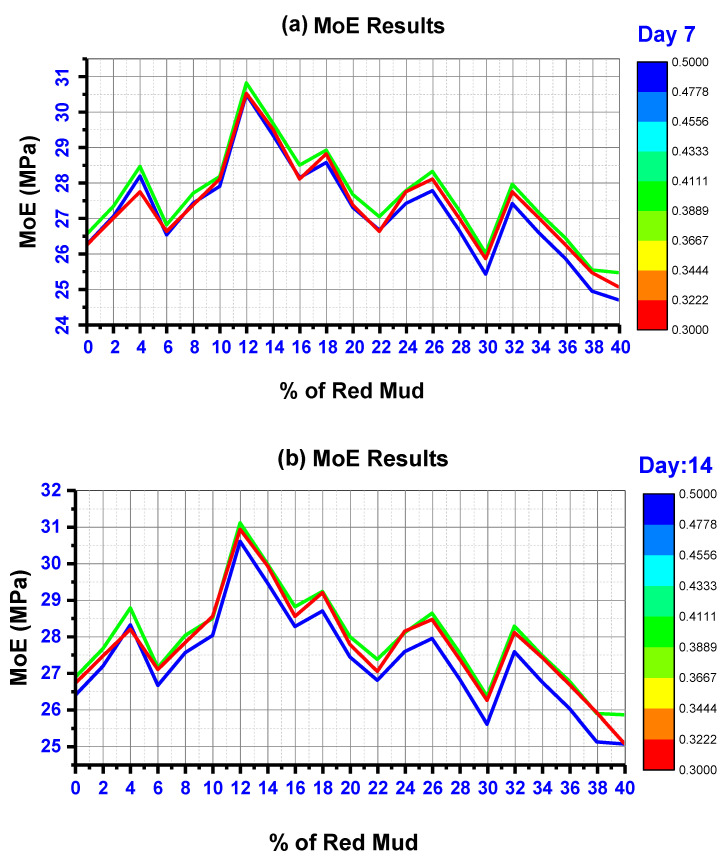

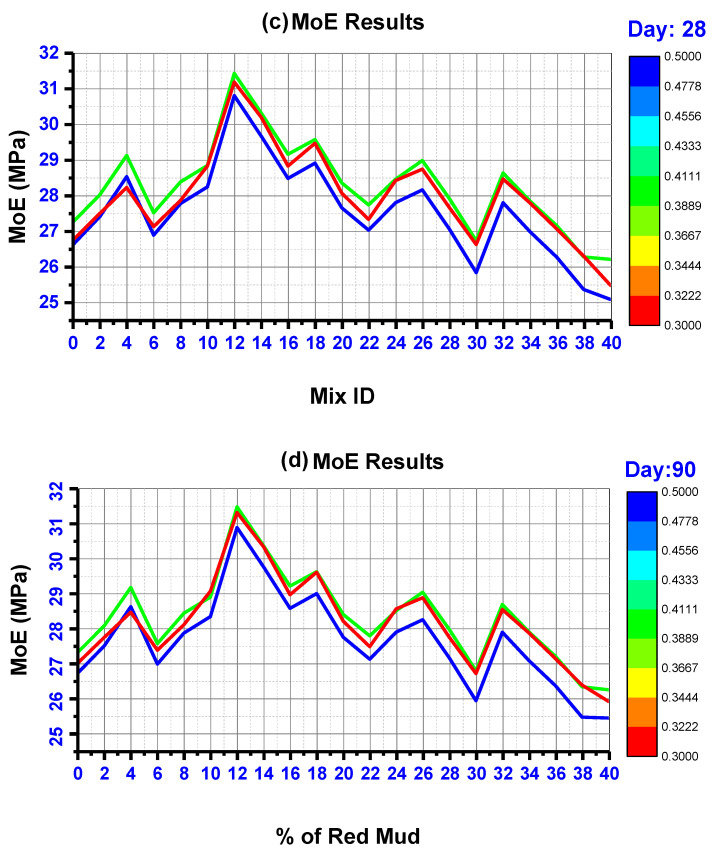
MoE results for various mix IDs for the day of (**a**) 7, (**b**) 14, (**c**) 28, and (**d**) 90. Red color indicates GPC matrix design for A/B ratio of 0.30, green color indicate GPC matrix design for A/B of 0.40, blue color indicate GPC matrix design for A/B ratio of 0.30.

The MoE test was performed according to IS 516 codes. Geopolymer cylinders were cast with the dimension of 150 mm in diameter and 300 mm in length for KOH and K_2_SiO_3_ activator agents. Figure 10 shows the MoE results of GPC for various A/B ratios tested for 7 to 90 days. A/B ratio of 0.40 shows excellent MoE results compared with other ratios. GR6 was the mix that showed maximum MoE, with 30.82 MPa for A/B of 0.40@ 7 day, 31.11 MPa for A/B of 0.40@ 14 day, 31.43 MPa for A/B of 0.40@ 28 day, 31.68 MPa for A/B of 0.40@ 90 day. As RM content increases, the MoE increases up to GR6 (maximum). Beyond this point, MoE starts to reduce. GR6 consists of 12% as RM. GR20 was found to have the lowest MoE value.

From Figure 10, it is clear that testing days have only a significant impact on the strength development of MoE. At 90 days of testing MoE, strength increment is less than 2% compared with other days of testing. Several factors influence the MoE of GPC, including the kind of binder used, the casting procedure, the type of activator used, the curing condition and temperature, etc. The implementation of the mix design approach has increased MoE [79,83]. Additionally, varying viscosities and alkaline silicates of different cations may impact processes throughout the geopolymerization process, resulting in diverse microstructures and mechanical properties. The microstructure of GPC materials made with potassium silicate was much denser (less porous) than that of sodium silicate matrixes [54,84].

#### 4.4.5. Impact Energy

**Figure 11:**
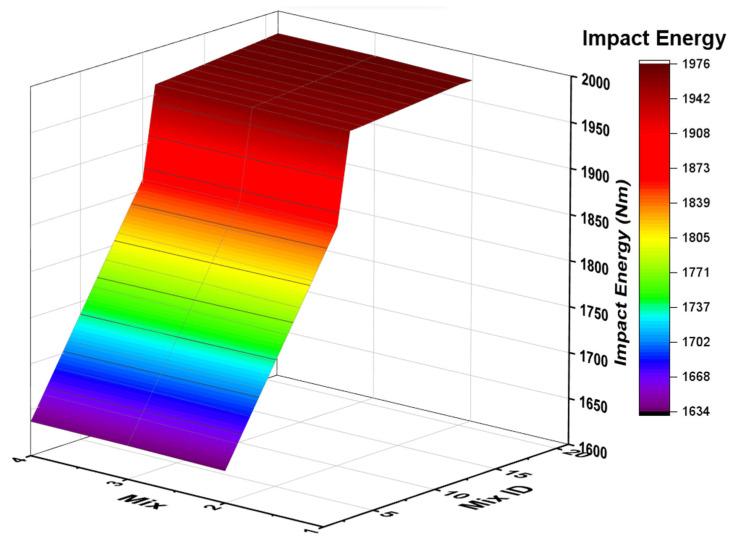
Three-dimensional impact tests plot for various A/B ratios. Red coloure indicates higher impact energy and yellow—medium whereas violet lower range.

Figure 11 shows the impact resistance results for various A/B ratios of 0.30, 0.40, and 0.50 for KOH and K_2_SiO_3_ as activator agents. At the increase of the A/B ratio, the impact value also increased. The impact value observed for the GPC series was in the range of 1600 to 2000 Nm.

The A/B ratio of 0.50 shows maximum impact value compared with other ratios. GR19 is the sample for which the maximum impact value of 1952 Nm was observed. From G0 to GR15, impact values showed an incremental relationship, but from GR16 to GR19, the impact value had very few marginal increments. GR19 had a maximum RM% compared with other proportions. For GR1 with A/B ratios of 0.30, it was recorded as a minimum. Cylindrical specimens were cast with a diameter of 50 mm and a length of 150 mm to study impact resistance. Impact resistance was studied using the dropping weight method. This entailed the following: dropping weight of 3 kg ball made up of steel with a height fall of 457 mm. The following equation was used in the computation of impact energy (Nm):

E = N ∗ m ∗ g ∗ h, wherein,

N is the number of strikes that cause the specimen to fall, 

m is the weight of the steel ball,

h is the freely falling height of the ball, and

g is the acceleration due to gravity.

#### 4.4.6. ANOVA

**Figure 12:**
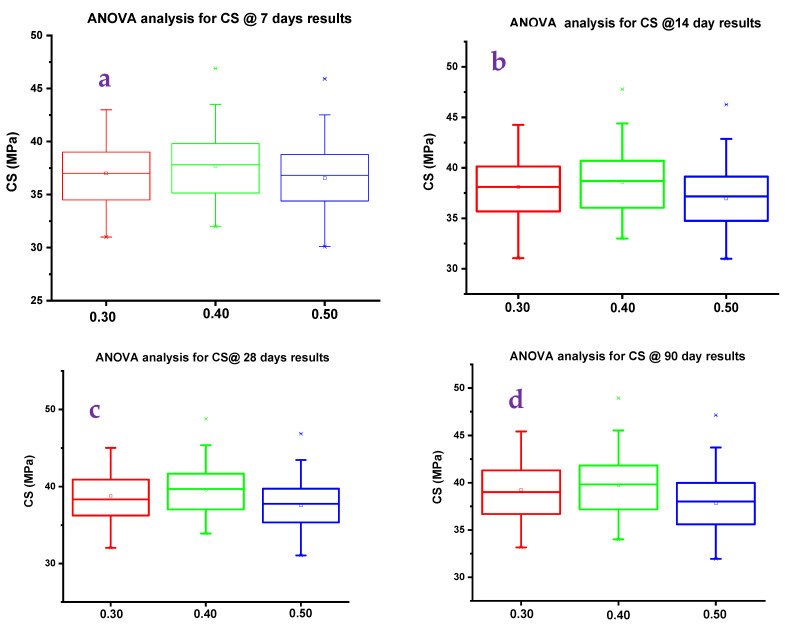
ANOVA analysis for various CS results of (**a**) 7 days, (**b**) 14 days, (**c**) 28 days, and (**d**) 90 days.

A/B ratio of 0.40 shows excellent CS results in all testing days from 7 to 90 days compared with other ratios. Figure 12 shows a one-way ANOVA analysis for CS results. The homogeneity of variance test used the Levene test for absolute deviation as the probability factor was 0.97156 for day 7 tested results. Similarly, the probability factor was 0.9919, 0.9879, and 0.9939 for 14, 28, and 90 days of testing, respectively. The Tukey test for CS results was used; the probability number obtained in ANOVA variation was 0.9067 for GPC tested for 7 days.

**Figure 13:**
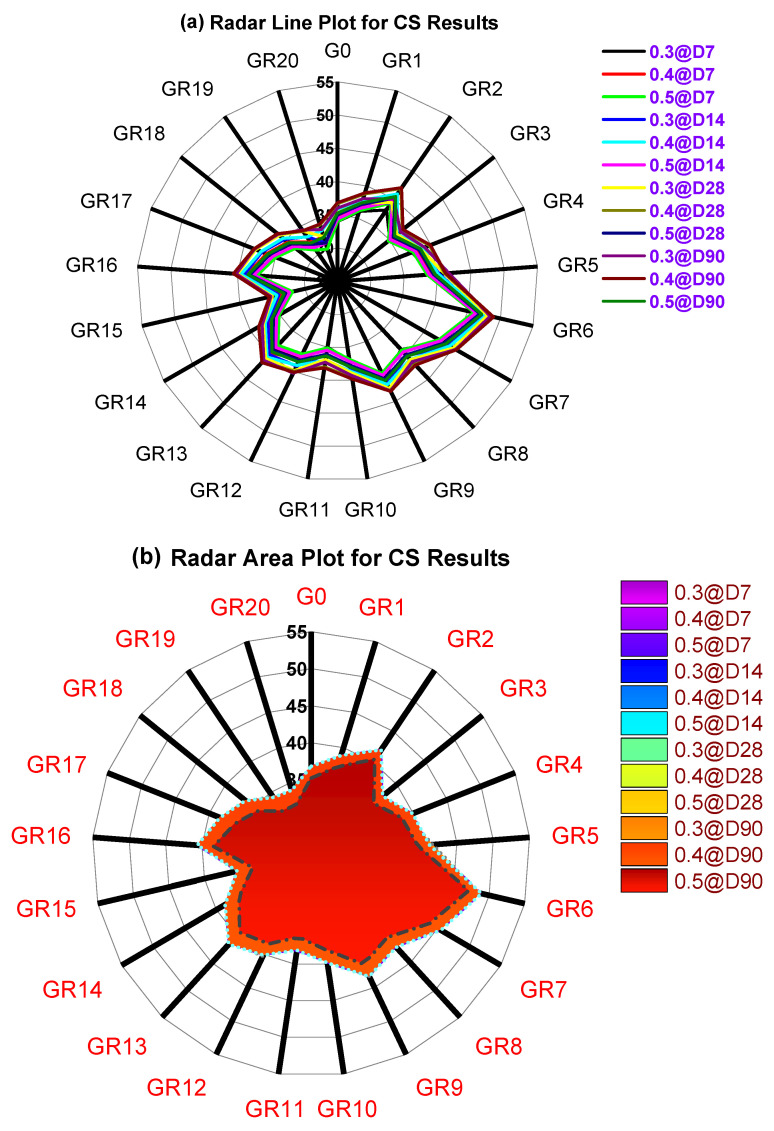
Radar plot for various CS results: (**a**) radar line plot and (**b**) radar area plot.

Similarly, the probability factor was 0.9067, 0.7676, and 0.9069 for 14, 28, and 90 days of testing, respectively. Levene and Tukey′s variation analysis showed an average probability of 92%, which indicates that the CS results line varies linearly. For an A/B ratio of 0.40, results show that CS results vary linearly for a testing period of 7 to 90 days. As the curing days progressed, the CS strength also increased. A 2 to 3% increment of CS value was observed from 7 to 14 days of testing for an A/B ratio of 0.40. For 28 and 90 days of testing, the variation of CS results for A/B ratios was 1 to 1.5% increment. The presence of RM as the binder in GPC showed incremental CS results, but for specimens tested for 28 and 90 days, the CS value increment was only marginal. Figure 13 shows the radar plot in line pattern (a) and area plot (b). GR6 shows the maximum CS value among all the mixes for an A/B ratio of 0.40 (refer to Figure 13b).

**Figure 14:**
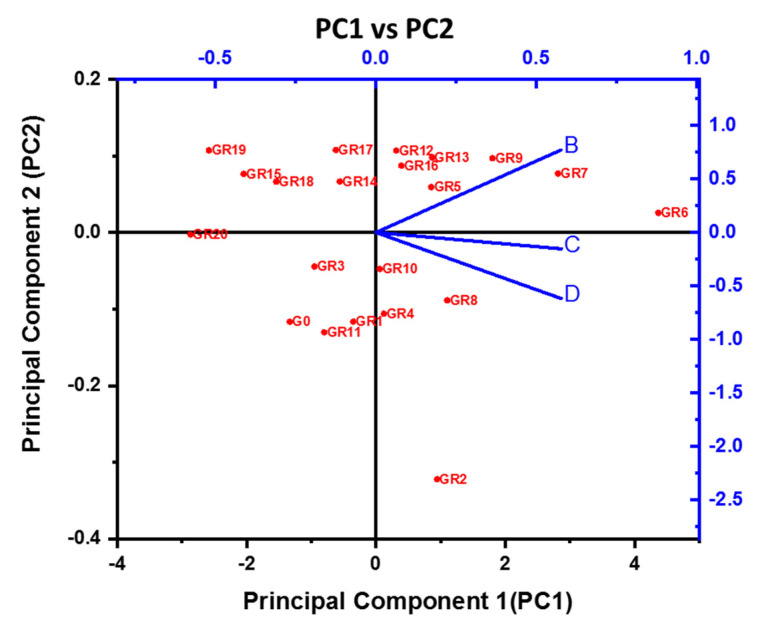
Principal component plot CS values.

Figure 14 shows the principal components plot for CS tested for 7, 28, and 90 days as D, C, and B components, respectively. GR6 shows the maximum principal component compared with other mixes. The B line indicates that the maximum CS value was obtained for 90 days of testing. The correlation matrix for B was 0.9875. Similarly, for C and D, the values were 0.9964 and 0.99, respectively. The correlation matrix indicates the regression (R2) of all the CS results. On average, CS values had a regression of 0.99.

Red color indicates various mix id used for current study, blue color indicate principal components scale. The compressive strength of geopolymer pastes using a Na-based activator was stronger at ambient temperature and higher at raised temperatures up to 40 °C than its K-based counterpart. The compressive strength of a geopolymer containing a K-based activator is somewhat greater than its Na-based equivalent at 60 °C. The geopolymer paste with a K-based activator had more significant residual compressive strength at increased temperature conditions than its Na-based equivalent. The geopolymer paste containing a K-based activator with a K_2_SiO_3_/KOH ratio of 3 had higher residual compressive strength at all increased temperatures than its Na-based equivalent.

## 5. Conclusions

The effect of temperature on the compressive strength parameter using destructive and non-destructive testing was analyzed. Some critical observations and conclusions are obtained as follows.

Fresh and mechanical results showed that materials of geological origin rich in silica and alumina content enhance the performance of geopolymer concrete. Replacement of ground granulated blast furnace (GGBS) with 12% RM showed a positive effect on the geopolymerization process. The addition of activator agents such as KOH and K_2_SiO_3_ increased the workability of geopolymer concrete up to 78 mm. The compressive strength of geopolymer concrete increased with age. For an alkali to binder ratio (A/B) ratio of 0.45 and RM of 12%, maximum compressive, tensile, flexural, modulus of elasticity (MoE), and impact strength were observed. Both Levene and Tukey′s variation analysis show a 92% average likelihood, indicating that the compressive strength (CS). CS results line varies linearly. The formation of calcium alumina silicate hydrate (C–A–S–H) gel was limited at greater RM replacement levels, resulting in a reduction in CS. The alkali fusion process dramatically improves the reactivity of red mud (RM) by geopolymerization. High silica and alumina-rich materials in the geopolymer result in a reduced polycondensation level of the geopolymer owing to inadequate dissolving and potential agglomeration of the materials, which has an unfavorable impact on the mechanical characteristics of the geopolymer.

## Figures and Tables

**Table 1:** Chemical composition of GGBS and RM.

Composition Binder	SiO_2_	Al_2_O_3_	CaO	MgO	K_2_O	Fe_2_O_3_	Na_2_O	SO_3_	Others	LOI
GGBS	34.80	15.78	36.81	7.09	0.44	0.38	0.27	2.53	-	1.50
RM	6.95	16.18	11.24	-	-	42.35	3.45	-	-	4.25

**Table 2:** Physical properties of ingredients of GPC.

Materials	GGBS	Red Mud	Aggregate	R. Sand
Physical Properties				
Specific gravity	2.88	2.68	2.8	2.6
Zone	-	-	-	II
Fineness modulus	-	-	7.0	3.0
Silt content	-	-	-	4%

**Table 3:** Mix proportion of GPC.

Mix ID	GGBS %	RM %	Fine Aggregate (kg/m^3^)	Coarse Aggregate (kg/m^3^)	Alkali/Binder Ratio	Alkaline Solution	
						KOH (kg/m^3^)	K_2_SiO_3_ (kg/m^3^)
G0	100	0	554	1295	0.30, 0.40, 0.50	14.66	52.4
GR1	98	2	554	1295		14.66	52.4
GR2	96	4	554	1295		14.66	52.4
GR3	94	6	554	1295		14.66	52.4
GR4	92	8	554	1295		14.66	52.4
GR5	90	10	554	1295		14.66	52.4
GR6	88	12	554	1295		14.66	52.4
GR7	86	14	554	1295		14.66	52.4
GR8	84	16	554	1295		14.66	52.4
GR9	82	18	554	1295		14.66	52.4
GR10	80	20	554	1295		14.66	52.4
GR11	78	22	554	1295		14.66	52.4
GR12	76	24	554	1295		14.66	52.4
GR13	74	26	554	1295		14.66	52.4
GR14	72	28	554	1295		14.66	52.4
GR15	70	30	554	1295		14.66	52.4
GR16	68	32	554	1295		14.66	52.4
GR17	66	34	554	1295		14.66	52.4
GR18	64	36	554	1295		14.66	52.4
GR19	62	38	554	1295		14.66	52.4
GR20	60	40	554	1295		14.66	52.4

## Data Availability

The data that support the findings of this study are available on request from the corresponding author, [SVG].

## Nomenclature/Abbreviations/Symbols

A/B	Alkali/Binder Ratio
Al	Alumina
ASTM	American Society for Testing and Materials
Al_2_O_3_	Aluminum oxide
CA	Calcium
CASH	Calcium Alumina Silica Hydrate
CE	Carbon Emission
CS	Compressive Strength
CTM	Compression Testing Machine
EE	Embodied Energy
FA	Fly Ash
GGBS	Ground Granulated Blast-Furnace Slag
GPB	Geopolymer Brick
IS	Indian Standard
K	Potassium
Kg	Kilogram
MPa	Mega Pascal
MJ	Mega Joule
Na	Sodium
Na_2_SiO_3_	Sodium Silicate
NaOH	Sodium Hydroxide
NASH	Sodium Alumina Silica Hydrate
O	Oxygen
RHA	Rich Husk Ash
Si	Silica
SiO_2_	Silicon Oxide
SiO_4_	Silicon Oxygen Tetrahedron
WA	Water Absorption
°C	Degree Celsius
